# Comparison of Trehalose/Hyaluronic Acid (HA) vs. 0.001% Hydrocortisone/HA Eyedrops on Signs and Inflammatory Markers in a Desiccating Model of Dry Eye Disease (DED)

**DOI:** 10.3390/jcm11061518

**Published:** 2022-03-10

**Authors:** Gloria Astolfi, Luca Lorenzini, Francesca Gobbo, Giuseppe Sarli, Piera Versura

**Affiliations:** 1 Laboratory for Ocular Surface Analysis and Translational Research, Dipartimento di Medicina Specialistica, Diagnostica e Sperimentale (DIMES), Alma Mater Studiorum—University of Bologna, 40138 Bologna, Italy; gloria.astolfi2@unibo.it; 2 Department of Veterinary Medical Sciences (DIMEVET), Alma Mater Studiorum—University of Bologna, Ozzano dell’Emilia, 40064 Bologna, Italy; luca.lorenzini8@unibo.it (L.L.); francesca.gobbo3@unibo.it (F.G.); giuseppe.sarli@unibo.it (G.S.); 3 Interdepartmental Centre for Industrial Research in Health Sciences and Technologies, CIRI Health Sciences and Technologies, Ozzano dell’Emilia, 40064 Bologna, Italy

**Keywords:** dry eye disease (DED), mouse model, desiccation stress, controlled-environment chamber, trehalose, sodium hyaluronate, corneal damage, mucins, inflammatory markers, hydrocortisone

## Abstract

Background: Dry eye disease (DED) is a multifactorial disease where ocular surface inflammation and damage play key etiological roles. Purpose: To compare a combination of 3% trehalose (T) and 0.15% hyaluronic acid (HA) (Thealoz duo^®^, T/HA) with a tear substitute containing 0.001% hydrocortisone (I) and 0.2% HA (Idroflog^®^, I/HA), with respect to changes on signs and inflammatory markers in a mouse DED model. Methods: Thirty 12-week-old C57BL/6 mice were exposed in a controlled-environment chamber as a desiccating stress model of DED for 35 days. At day 14 (T1), administration of 5 µL T or I in the right eye (RE) or NaCl 0.9% in the left eye (LE) started, twice a day. Animals were sacrificed after 7 (T2), 14 (T3), 21 (T4, endpoint) days from the beginning of treatment. Corneal fluorescein staining ratio (Image J), histological and histochemical assessment of ocular surface tissues (goblet cell GC density and characterization —PAS, Alcian blue pH 2.5, pH 1.0, and MUC4 expression—in the superior and inferior conjunctiva), and levels of inflammatory markers HLA-DR, IL-1β and TNF-α in cornea and conjunctiva were measured. Results: No animal fully recovered from DED signs at the endpoint. Difference between arms was observed at T3 and T4, with T treated eyes showing a higher corneal damage reduction, PAS-positive GC recovery, lower inflammatory marker expression as compared to the I treated ones. Conclusions: Data suggest that 21 days of treatment with T/HA improved signs, GC recovery and inflammatory markers in a DED mouse model, to a greater extent as compared to I/HA. Data suggest that 21 days of treatment with T/HA improved signs, GC recovery and inflammatory markers in a DED mouse model, to a greater extent as compared to I/HA.

## 1. Introduction

Dry eye disease (DED) is now recognized as a multifactorial chronic inflammatory disorder, which results in symptoms of ocular discomfort, tear film instability and hyperosmolarity, visual disturbance, and in which neurosensory abnormalities play etiological roles [1]. DED is one of the most common reasons for seeking medical care across the world [2] and is a growing global public health problem [3]. It represents a considerable burden either for the patient, impacting the quality of life [4] and psychological impact of pain [5], as well as for society with direct and indirect costs due to healthcare system charge and loss of work productivity, respectively [6]. The prevalence of DED ranges from 5% to 50%, depending on age, sex, and geographical area [7].

The tear film evaporative water loss and instability leading to hyperosmolarity, and tissue damage represent the core mechanism of DED [8,9]. Research in humans and in animal models has shown that this occurrence triggers inflammatory cascades leading to further cell death, including loss of mucin-producing conjunctival goblet cells, which exacerbates tear film instability and perpetuates this loop of events [10]. Numerous inflammatory cytokines are elevated in the tear film of patients with DED [11,12] and clinical inflammatory signs are used to diagnose and grade the severity of DED in daily practice [13]. A variety of treatments targeted to avoid or delay DED progression are proposed with the rationale to break at any point the vicious circle of ocular surface inflammation, driven by both innate and adaptive immune response mechanisms [13,14].

Trehalose is a natural nonreducing disaccharide, widespread in many species of desiccation-tolerant plants and invertebrate animals, which stabilizes protein and membranes [15,16], and also exhibits bio- and osmoprotectant roles [17], it mitigates the effects of desiccation on corneal cells [18] and reduces levels of inflammatory cytokines in DED animal models [19] and in patients [20].

In this study, we aimed to deepen the knowledge on the effect of trehalose on clinical signs and inflammatory markers, which are both increased in DED. This was accomplished on a DED desiccating mouse model, by treating animals with a commercially available tear substitute containing trehalose and having as a comparator a product with an extremely low percentage of steroid, marketed as a tear substitute as well, and both containing hyaluronic acid.

## 2. Materials and Methods

### 2.1. Animals

Female, pathogen-free, twelve-week-old, C57BL/6 mice (Charles River, Calco, Italy) were used in these experiments. Both eyes were used to reduce the number of animals. Mice were handled in accordance with the ARVO Statement for the Use of Animals in Ophthalmic and Vision Research (TS 12/14). All animal protocols described were supervised by an expert in laboratory animal healthcare and carried out according to the European Community Council Directives 2010/463/UE, approved by the Italian Ministry of Health (D. Lgs 26/2014, authorization n°724/2020-PR, 21 July 2020). Moreover, animal protocols were carried out in compliance with the guidelines published in the ARRIVE and NIH Guide for the Care and Use of laboratory animals.

### 2.2. DED Model

**Figure 1:**
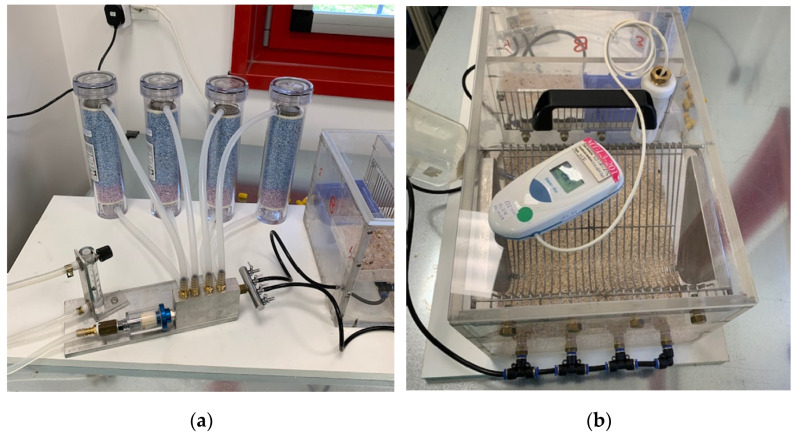
(**a**,**b**)—Figure of the induced dry eye disease model (**a**) System with air pump, water separator, desiccators, and flowmeter with valve; (**b**) Cage with temperature—humidity recorder and probe.

Induction of the DED Model requires a Controlled-Environment Chamber (CEC) that guarantees specific conditions of airflow, humidity and temperature, as previously described [21]. The usable floor area of our CEC is 800 cm^2^. The roof of the cage is sealed with isolating material and one hole on the roof allows the air to move outside. For desiccants, we use anhydrous CaSO_4_ (Drierite; W.A. Hammond Drierite Co., Xenia, OH, USA). As a source of air, we use a small, low-noise (38 dB) oilless linear pump (Enviro 40 L/min open flow, 30 W; Charles Austen Pumps Ltd., West Byfleet, UK). The airflow is regulated by a flowmeter. The air is pumped into the chamber through four tips (1 mm diameter) placed in two opposite walls (four per wall) (Figure 1a,b). The height of the tips has been chosen to correspond to the height of the mouse’s eyes. The chamber was placed in a room with temperature maintained at 21 °C to 23 °C, at a relative humidity (RH) of 40% to 60%. Parameters were recorded over the entire period of study.

### 2.3. Experimental Design

For dry eye induction, mice were placed into the CEC and exposed for 12 h/day (9.00 p.m. to 9 a.m.) to controlled environmental conditions (RH, <25%; AF, 8 L/min; temperature, 21–23 °C) for 14 days. During all phases of experimentation food and water were provided *ad libitum*. Before exposure to CEC, the cornea of both eyes was assessed by instillation of fluorescein and observed using a cobalt blue light. Mice with severe corneal damage prior to DED induction were not included in experimentation and replaced.

Pre-injury evaluations (T0) were performed to check the corneal integrity, then the animals were randomly divided into experimental groups and organized into treated mice (*n* = 30) exposed to the CEC chamber and non-exposed (*n* = 6). Five animals were included per group/time point.

Mice were placed in the CEC for 12 h/day for a period of 14 consecutive days (T1). Six control animals were age-matched mice kept in a standard cage with a normal environment (RH 50–80%, no AF, T 21–23 °C), and these animals will be defined in the study as Wild Type (WT) controls.

Deeply isoflurane-anesthetized animals were euthanized after 7 (T2), 14 (T3), and 21 (T4, endpoint) days from the beginning of treatment. The eye globes, including both superior and inferior lids, were excised, washed in cold PBS pH 7.4 to remove the excess of blood, and tissues were further processed as described below.

### 2.4. Treatments

Treatment was always carried out on the right eye (RE) of each animal. We tested two different preservative-free eye drops, one based on the combination of 3% trehalose and 0.15% sodium hyaluronate (commercially available as Thealoz Duo^©^, Thea Pharma, Laboratories Thea, Clermont-Ferrand, Cedex 2, France) and the other based on 0.2% sodium hyaluronate and 0.001% hydrocortisone (commercially available as Idroflog^©^, Alfa Intes, Casoria, Naples, Italy). One group (*n* = 15, arm A, T/HA) received the treatment with Thealoz Duo^©^ eye drops, the other group (*n* = 15, arm B, I/HA) was treated with Idroflog eye drops. The left eye (LE) acted as a control and was treated with a drop of saline solution (0.9% NaCl).

Treatments for the two groups were applied with one drop two times a day (9.00 am and 5.00 pm) and continued for 7 (T2), 14 (T3), and 21 (T4) days while animals were still subjected to environmental stress induction.

### 2.5. Corneal Fluorescein Staining

The corneal integrity was evaluated and photographed at T1, T2, T3, T4, in isoflurane sedated mice, with a slit lamp biomicroscope using a cobalt blue light 3 min after the application of 0.5 µL of 0.2% fluorescein (Fluoralfa, Alfa Intes, Casoria, Naples, Italy), using micropipette into the inferior conjunctival sac of the right eye (RE) and left eye (LE), and the excess liquid was absorbed from the lateral canthus. Detection and quantification of fluorescein-stained areas (‘‘stained pixels’’) for the correlation analysis, the corneal staining score was calculated.

The images were analyzed using the public domain software Image J (v1.53a, National Institutes of Health, Bethesda, MD, USA), an open-source image processing/analysis software. The corneal staining ratio (CSR), defined as the ratio between the staining area and the total corneal area, was calculated and expressed as a percentage [22].

### 2.6. Histological Analysis

The specimens assigned to morphological analyses were fixed in 4% buffered formaldehyde and stored at room temperature until processing and were then dehydrated in an ascending series of ethanol and included in paraffin. Four 3-µm-thick sections obtained from the central vertical plane of the ocular globe were stained with haematoxylin and eosin to evaluate conjunctival epithelial morphology and with periodic acid-Schiff (PAS) (cat no. 04-130802, Bio-Optica, Milan, Italy), Alcian pH 2.5 (cat no. 04-160802 Bio-Optica, Milan, Italy) and Alcian pH 1 (HCl 0.1N 1% Alcian Blu, cat no. CA8GX-P-5, Histo-Line Laboratories, Pantigliate, Milan, Italy) respectively to ascertain changes in neutral mucins (PAS), acid non-sulfated mucins (Alcian pH 2.5) and acid sulphated mucins (Alcian pH 1) in GCs.

GCs count was performed on images acquired with an optical microscope (Eclipse E600; Nikon, Shinjuku, Japan) equipped with the Imaging Source “33” Series USB 3.0 Camera (cat no. DFK 33UX264; Bremen, Germany), at 250× magnification. Three different sections were selected for counting GCs, in both superior and inferior lids and a median of both areas was calculated/100 µm length.

### 2.7. Immunohistochemistry MUC 4

Formalin fixed-paraffin embedded 3-µm-thick sections were labeled with an LSAB kit (DAKO, Santa Clara, CA, USA) and anti-mucin-4 antibody (Invitrogen, Waltham, MA, USA, Cat# PA5-23077, 1:1000). Sections were visualized with a DAB substrate kit (Histo-Line Laboratories, Milan, Italy), and observed under an Olympus BX51 microscope. The signal intensity of mucin-4 was measured using Image J software v.1.51j8 (NIH, Bethesda, MD, USA) [22].

### 2.8. RNA Extraction and cDNA Reverse Transcription

For the molecular biology analyses, the excised samples were sectioned to further separate the cornea and conjunctiva from the eye globe. Total RNA extraction was performed by using TRIreagent (TRIzol reagent, Thermo Fisher Scientific, Waltham, MA, USA) according to the manufacturer’s instructions. Phenol-chloroform extraction procedure was used for total RNA extraction from dissected ocular tissues. RNA was precipitated with isopropanol and washed in ethanol before reconstitution in nuclease-free water. Two-microliter RNA samples were spectrophotometrically analyzed, and optical density was recorded according to a standard procedure (A1000 Nanodrop, Celbio, Milan, Italy) and one μg of total RNA was reverse transcribed in a 20 μL reaction volume using the iScriptTM cDNA synthesis kit (BioRad Laboratories, Hercules, CA, USA) following the manufacturer’s instructions.

Real-Time PCR analysis was carried out on a QuantStudio™ 5 Real-Time PCR System (Applied Biosystems^®^ by Life Technologies, Carlsbad, CA, USA), using the semi-quantitative Sybr Green (Sso AdvancedTM Universal Sybr Green Supermix; BioRad Laboratories, Hercules, CA, USA) approach.

The assay was executed in triplicate and target gene expression was normalized to β-actin (mouse) was used as a housekeeping gene. The final results were determined by the comparative 2^−ΔΔCt^ method and expressed as fold changes relative to WT. Specific couples of primers were designed by using the NCBI Blast Tool (Table 1).

### 2.9. Statistical Analysis

The Mann–Whitney unpaired test for corneal fluorescein staining and GC count was used to compare the differences between the T/HA group, I/HA group and the wild type control eyes. A paired *t*-test would be applicable. The paired *t*-test and the Wilcoxon test were used to compare the study and control eye (of the same mouse) and the within-group changes from baseline. All tests were two-tailed, and a *p* < 0.05 was considered to be statistically significant.

## 3. Results

### 3.1. Animal Wellness

Animals living in the CEC demonstrated normal behavior, and no manifestation of stress responses (e.g., weight loss, excessive eye rubbing, fur loss, aggressive behavior, mutilation) was noted over the whole period of observation. The weight gain and the food and water intake of the mice living in the CEC and in the control groups were similar during the period of the study (median weight at T0 16.9 ± 0.2 g in both groups; at T4 19.1 ± 0.2 g in CEC group, and 19.0 ± 0.1 in the control group).

### 3.2. DED Model

During the whole period of desiccating stress induction, the recorded temperature was 22.5 ± 0.2 °C, similar to the room temperature. The overall mean humidity in the CEC was 20.5 ± 4.1%, The air flux was kept constant at 8 L/min.

**Figure 2:**
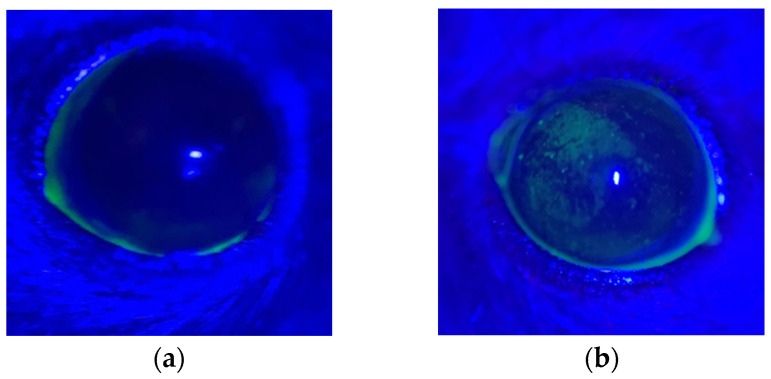
(**a**) Control animal pre-DED induction, baseline (T0), left eye; (**b**) Animal stressed for 14 days in the CEC (T1), right eye. A micropuntate fluorescein positive corneal damage is observed throughout the cornea, especially in the upper nasal quadrant.

Minimal punctuate corneal fluorescein staining was observed at baseline (T0) in both the wild type control and the study groups (4.9 ± 1.2%), without significant differences between the two groups (Figure 2a). A statistically significant increase in staining was recorded in the CEC-living mice at day 14 (21.5 ± 3.5%, *p* < 0.05 versus T0, Figure 2b), whereas in the control group no difference was recorded versus T0.

### 3.3. Corneal Damage—Fluorescein Staining-Effect of Treatment

**Figure 3:**
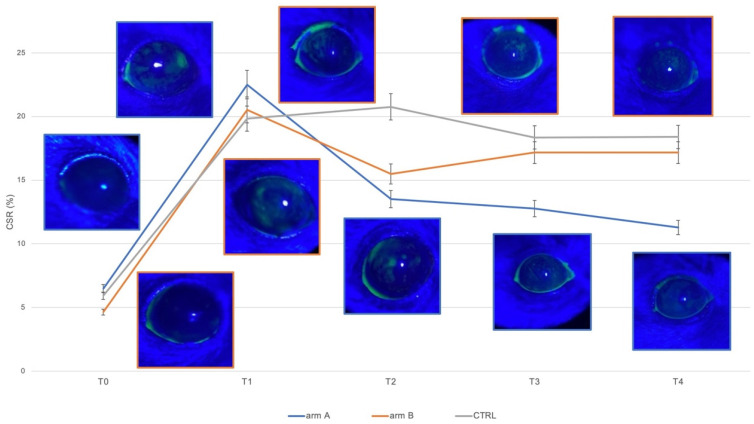
Right eye (treated with the two products under study) analysis of corneal staining ratio at different points: control pre-DED induction (T0); after a period of 14 days of induction DED (T1); after 7 days (T2), after 14 days (T3), and after 21 days (T4) of treatment with Thealoz Duo^®^ (arm A, blue line) or with Idroflog^®^ (arm B, orange line). Data are graphed as median and 5% error interval. Representative images for each point and each arm are included in the figure, surrounded by the corresponding colors defining the two arms. Left eye (treated with 0.9 % NaCl in both arms) analysis is graphed with the grey line at the same time points, this series acts as a neutral control. CSR = Corneal staining ratio, expressed as a percentage and calculated as the ratio between the stained corneal area and the total corneal area.

The CSR at T1 was the same in the animals of both arms with no significant differences between the eyes. After 7 days of treatment (T2), CSR showed a statistically significant reduction as compared to T1 only in the right eyes (*p* < 0.001), but without significant differences between arms. In the left eyes treated with 0.9% NaCl, no change in CSR was observed throughout the entire study as compared to T1 (*p* always > 0.05). The CSR observed at T3 and T4 appeared progressively reduced compared to T2 (*p* < 0.05) and compared to T1 (*p* < 0.0001) only in the right eyes of the animals of arm A, reaching a reduction by the half at the endpoint. The CSR observed in the right eyes of the animals of arm B showed values similar to those recorded at T2 (*p* not significant). Data are graphed in Figure 3.

No animal fully recovered from corneal damage at the endpoint, but a statistically significant difference in CSR was found between arms (*p* < 0.0001).

### 3.4. Histologic Analysis

**Figure 4:**
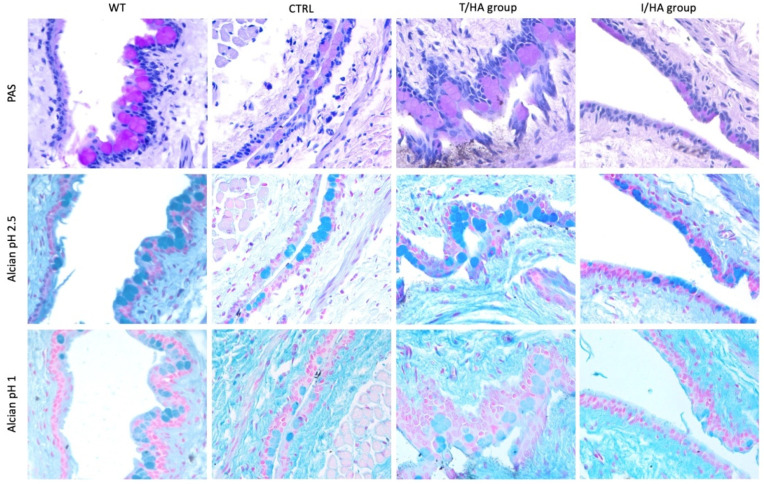
Representative images of the conjunctival epithelium of Wild Type (WT) animals (those not living in CEC, left column) and treated animals (those living in CEC) at T4 (21 days of treatment, endpoint) (all the other columns). In particular: LE (Left Eye) of animals living in CEC (CTRL, middle left column), RE (Right Eye) of animals belonging to T/HA group (middle right column), RE of animals belonging to I/HA group (right column). PAS-positive GCs (upper row, pink round cells), Alcian Blue pH 2.5 positive GCs (middle row, dark blue cells), Alcian Blue pH 1 positive GCs (bottom row, light blue cells). Light microscopy, 400× original magnification.

The GCs were found distributed in groups and intercalated within the epithelial cells of the conjunctiva in all the samples, with different densities, as shown in representative images collected in Figure 4 and summarized in Table 2. PAS, Alcian Blue pH 2.5 and 1 positive GCs appeared evenly distributed in the conjunctival epithelium of the WT animals, and a statistically significant reduction was observed in both eyes of the CEC stressed animals (Figure 4).

In particular, the CEC stressed animals showed a higher reduction of all the GCs in the LE (CTRL) treated with 0.9% NaCl (*p* < 0.001). A statistically significant difference between Arms was found only for neutral mucin expression (PAS-positive GCs) (*p* < 0.01), with a partial recovery of GC density in Arm A animals (Table 2).

### 3.5. Immunohistochemistry for MUC4

MUC4 positivity was found at the apical portion of the conjunctival epithelial cells in WT animals, with an apparent diminution focally found in CEC stressed animals. Data are extremely animal dependent, and no consistent result could be drawn on the effect of treatments on MUC4 expression.

### 3.6. Molecular Biology for Inflammatory Marker (HLA-DR, IL-1β, TNF-α) Expression

**Figure 5:**
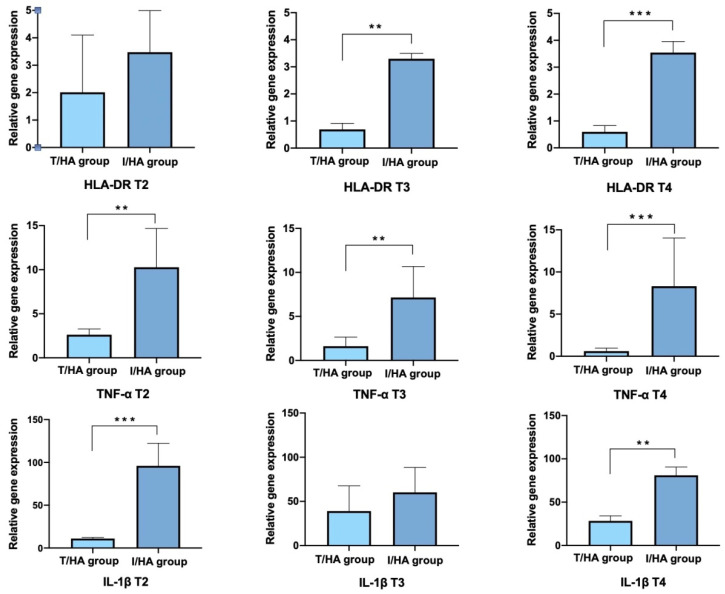
Results for HLA-DR (upper row), TNF-α (middle row), and IL-1β (bottom row) expression in RE treated for 7 (**left column**), 14 (**middle column**), and 21 (**right column**) days with the two products under study. Arm A = 3% trehalose/0.15% HA (T/HA), light blue; Arm B = 0.001% hydrocortisone/0.2% HA (I/HA), dark blue. Significance ** *p* < 0.001; *** *p* < 0.0001.

The expression of all the inflammatory markers increased as a consequence of desiccation stress induced by CEC as compared to WT control animals, in both eyes. After desiccation stress (T1) the increase of expression was 5.5-fold for HLA-DR, 10.3-fold for TNF-α, and 80.5-fold for IL-1β, as compared to the WT expression (T0), in LE. A statistically significant lower expression of TNFα was found in the eyes of arm A animals with respect to arm B ones, starting from T2, and constantly remaining at the same level at T3 and T4. HLA-DR expression was found to be statistically significantly lower in the eyes of arm A animal starting from T3, and remaining at the same levels at T4. IL-1β expression showed a statistically significant difference between arms only at T2 and T4 (Figure 5).

## 4. Discussion

The rationale of current therapeutic options for DED is to break down the self-perpetuating cycle leading to a progressive worsening and to relieve subjective symptoms, although often this occurs only temporarily. Data from this study showed that the administration of a Trehalose-containing tear substitute in a DED mouse model improved clinical signs, GC recovery and reduced inflammatory markers, thus suggesting a breaking effect on the cycle.

Our work utilized a mouse model for DED induction which utilizes desiccating stress to induce significant alterations in GC density, and DED-related ocular surface signs [21], mucin expression decrease [23], and the involvement of innate immune system pathways with the predominance of pro-inflammatory cytokines, in particular IL-1β and TNF-α. [24]. This is believed a suitable model in mimicking the environmental stressful conditions the subjects experience in daily life [25], and our model was designed on the continuous induction of stress throughout the study. Three weeks of treatment might be considered insufficient to mimic what happens in real life, but the duration of the study was estimated to ethically balance scientific needs and induced animal suffering and pain, which was confirmed by the overall well-being of the animal at any stage, and in agreement with several other studies [26].

Our DED model was effective in causing corneal damage, reduced GC density and mucin changes, increased levels of specific inflammatory cytokines, in agreement with other models of desiccating stress. The comparator compound used in the other arm for this study was tailored on another tear substitute containing low dose corticosteroid and registered as a medical device, both containing a similar concentration of sodium hyaluronate. It has been hypothesized that the comparator compound might help to prevent the risk of any recurrence of inflammatory events thanks to the ancillary action of the corticosteroid agent with a low dosage, mild anti-inflammatory and short duration of action [27,28,29]. Data available in the previous literature are not comparable to our results, either for different DED models used [28] or for analytes evaluated [27,29], but taken as a whole would demonstrate an anti-inflammatory effect in the short term.

In the present study, no animal recovered completely from the induced corneal damage, as could be expected from the induction of desiccative stress concurrent with the administration of treatments. Interestingly, the effect of trehalose-based treatment on the reduction of corneal damage also demonstrated in previous works [20,30,31,32], was shown at 7 days. This can be due to the protective effects exerted by trehalose on the cell membrane, as shown in consistent previous results [17,31]. However, the reduction in corneal damage was reduced by half at the endpoint with the treatment with Trehalose-based eyedrops, whereas with the low-dose steroid the reduction was significantly less, remaining the comparator at essentially stable values throughout the duration of the study.

The distribution pattern of GCs within the conjunctival epithelium differs between humans and mice and is species-specific [33]. A common feature of all forms of DED is GC loss [8], and this decreased GC density is proportional to the disease severity [34]. Both secreted and membrane-associated mucins play key roles in the ocular surface wettability and lubrication [35] and contribute differently to its protection [36]. Gel-forming mucin MUC5AC is secreted by the conjunctival GCs and contributes to tear film stability by binding and gelling water molecules, thus increasing water retention on the ocular surface for a longer time [33]. Reduction of GCs is a common finding in DED, and recovery of their density is a target for DED therapy [37]. The present study showed a statistically significant decrease of PAS-positive (neutral mucins) and Alcian blue pH 2.5 and pH 1 positive (respectively acid non-sulfated and acid sulfated mucins) GCs as a consequence of desiccation stress in agreement with others [38], on the same mouse strain. At the endpoint, the recovery of neutral mucin GC density was greater in eyes treated with trehalose-based eyedrops than in the comparator and in the saline-treated eyes, a finding confirming what was already observed in patients [20].

Membrane-associated MUC4 is most prevalent in conjunctival epithelium, contributing to the glycocalyx formation and in the maintenance of the mucosal barrier integrity [36]. MUC4 expression was shown previously significantly reduced in the conjunctival epithelium of DED patients as compared to normal subjects [39]. Data from this study are unfortunately not consistent on MUC4 expression, and this is mainly due to the large variability of data collected in animals. However, the recovery effect of trehalose on MUC4 expression was not shown previously on DED patients as well [20], not explored in the literature for the comparator, and further data are needed to deepen this aspect.

DED patients experience an episodic exacerbation of discomfort symptoms, followed by elevation of inflammation [40]. Inflammatory mediators, notably chemokines and cytokines, increase in ocular surface tissues and tears of animal models and DED patients and are now considered as a hallmark of the disease [11,41]. The evaluation of the IL-1β and TNF-α was performed in this study, being among the most representative cytokines in DED patients. HLA-DR is a transmembrane heterodimer belonging to the major histocompatibility complex (MHC) class II receptors, expressed in ocular surface cells, and considered a consistent biomarker of DED severity to be utilized in clinical trials [41,42,43].

In our model, we observed a significant increase of these proinflammatory markers in animals subjected to CEC stress as compared to those not stressed, in agreement with others [26,44]. As expected, treatment with saline as a simple washing was ineffective, and interestingly a reduction was observed with the two treatments, notwithstanding both do not qualify as drugs. The reduction for all the three proinflammatory markers was found to a significantly greater extent in the ocular surface of animals treated with trehalose-based eyedrops, at the endpoint after 21 days of treatment, and confirms previous results in DED patients [20] and mouse models [19] on several other proinflammatory analytes, including IL-2, IL-6, IL-17, MMP-9, among others. As inflammatory mediators negatively impact GC differentiation, proliferation, and ultimately mucin secretion [37,45], the reduction observed may have had contributed to the concurrent GC recovery.

In conclusion, since inflammation plays a pivotal role in the pathogenesis and self-sustaining of dry eye disease, a therapy targeting the modulation of inflammation is required. In this task, natural molecules such as trehalose could play an important role in long-term therapies without adverse side events.

## Figures and Tables

**Table 1:** List of primer sequences used for Real-Time PCR.

Gene	Forward	Reverse
β-actin	GCAAGCAGGAGTACGATGAGT	AGGGTGTAAAACGCAGCTCAG
TNF-α	ACTGAACTTCGGGGTGATCG	TGGTGGTTTGTGAGTGTGAGG
HLA-DR	TTTACGACTGCAGGGTGGAG	AGGGCTTGGAGCATCAAACT
IL-1β	TGCCACCTTTTGACAGTGATG	TTGGAAGCAGCCCTTCATCTT

IL-1β: interleukin 1 Beta; TNF-α: tumor necrosis factor α; HLA-DRA: Major Histocompatibility Complex, Class II, DR.

**Table 2:** GCs count in Wild Type (WT) animals (those not living in CEC) and treated animals (those living in CEC) at T4 (21 days of treatment, endpoint). In particular: LE of animals living in CEC (CTRL), RE of animals belonging to T/HA group, RE of animals belonging to I/HA group. The density of PAS, Alcian Blue pH 1, Alcian Blue pH 2.5 positive GCs was expressed as Median (CI 95%).

Goblet cells	PAS	Alcian pH 1.0	Alcian pH 2.5
WT	9.4 (7.1–12.0)	8.4 (6.2–10.4)	8.9 (6.4–12.7)
LE-CTRL	4.0 (3.4–6.0) *	3.6 (2.5–5.6) *	5.5 (2.9–6.9) *
	[3.4–6.5]	[2.6–5.6]	[3.2–6.8]
RE-T/HA group	6.8 (4.2–8.9) *^,§^	4.8 (2.5–8.4) *	5.4 (2.1–6.9) *
	[6.0–9.1]	[2.6–8.1]	[2.3–6.9]
RE-I/HA group	4.7 (3.5–6.9) *^,§^	4.5 (3.1–5.2) *	6.0 (4.5–8.5) *
	[3.5–6.7]	[3.2–5.2]	[4.5–8.4]

* *p* < 0.001 significance versus WT; ^§^ *p* < 0.01 significance between arms.

## Data Availability

The data presented in this study are available upon well motivated request from the corresponding author.

## References

[1] Craig J.P., Nichols K.K., Akpek E.K., Caffery B., Dua H.S., Joo C.-K., Liu Z., Nelson J.D., Nichols J.J., Tsubota K. (2017). TFOS DEWS II Definition and Classification Report. Ocul. Surf..

[2] Bradley J.L., Stillman I., Pivneva I., Guerin A., Evans A.M., Dana R. (2019). Dry eye disease ranking among common reasons for seeking eye care in a large US claims database. Clin. Ophthalmol..

[3] Morthen M.K., Magno M.S., Utheim T.P., Snieder H., Jansonius N., Hammond C.J., Vehof J. (2021). The vision-related burden of dry eye. Ocul. Surf..

[4] Uchino M., Schaumberg D.A. (2013). Dry Eye Disease: Impact on Quality of Life and Vision. Curr. Ophthalmol. Rep..

[5] Belmonte C., Nichols J.J., Cox S.M., Brock J., Begley C.G., Bereiter D.A., Dartt D.A., Galor A., Hamrah P., Ivanusic J. (2017). TFOS DEWS II pain and sensation report. Ocul. Surf..

[6] McDonald M., Patel D.A., Keith M.S., Snedecor S.J. (2016). Economic and Humanistic Burden of Dry Eye Disease in Europe, North America, and Asia: A Systematic Literature Review. Ocul. Surf..

[7] Stapleton F., Alves M., Bunya V.Y., Jalbert I., Lekhanont K., Malet F., Na K.-S., Schaumberg D., Uchino M., Vehof J. (2017). TFOS DEWS II epidemiology report. Ocul. Surf..

[8] Bron A.J., de Paiva C.S., Chauhan S.K., Bonini S., Gabison E.E., Jain S., Knop E., Markoulli M., Ogawa Y., Perez V. (2017). TFOS DEWS II pathophysiology report. Ocul. Surf..

[9] Aggarwal S., Galor A. (2018). What’s new in dry eye disease diagnosis? Current advances and challenges. F1000Research.

[10] Baudouin C., Aragona P., Messmer E.M., Tomlinson A., Calonge M., Boboridis K.G., Akova Y.A., Geerling G., Labetoulle M., Rolando M. (2013). Role of Hyperosmolarity in the Pathogenesis and Management of Dry Eye Disease: Proceedings of the OCEAN Group Meeting. Ocul. Surf..

[11] Roda M., Corazza I., Reggiani M.L.B., Pellegrini M., Taroni L., Giannaccare G., Versura P. (2020). Dry Eye Disease and Tear Cytokine Levels—A Meta-Analysis. Int. J. Mol. Sci..

[12] Lam H., Bleiden L., de Paiva C., Farley W., Stern M.E., Pflugfelder S.C. (2009). Tear Cytokine Profiles in Dysfunctional Tear Syndrome. Am. J. Ophthalmol..

[13] Baudouin C., Irkeç M., Messmer E.M., Benítez-Del-Castillo J.M., Bonini S., Figueiredo F.C., Geerling G., Labetoulle M., Lemp M., Rolando M. (2017). Clinical impact of inflammation in dry eye disease: Proceedings of the ODISSEY group meeting. Acta Ophthalmol..

[14] Jones L., Downie L.E., Korb D., Benitez-Del-Castillo J.M., Dana R., Deng S.X., Dong P.N., Geerling G., Hida R.Y., Liu Y. (2017). TFOS DEWS II Management and Therapy Report. Ocul. Surf..

[15] Stolz J., Luyckx J., Baudouin C. (2011). Trehalose: An intriguing disaccharide with potential for medical application in ophthalmology. Clin. Ophthalmol..

[16] Koshland D., Tapia H. (2019). Desiccation tolerance: An unusual window into stress biology. Mol. Biol. Cell.

[17] Cejka C., Kubinova S., Cejkova J. (2019). Trehalose in Ophthalmology. Histol. Histopathol..

[18] Hovakimyan M., Ramoth T., Löbler M., Schmitz K.-P., Witt M., Guthoff R., Stachs O. (2012). Evaluation of Protective Effects of Trehalose on Desiccation of Epithelial Cells in Three Dimensional Reconstructed Human Corneal Epithelium. Curr. Eye Res..

[19] Li J., Roubeix C., Wang Y., Shi S., Liu G., Baudouin C., Chen W. (2012). Therapeutic efficacy of trehalose eye drops for treatment of murine dry eye induced by an intelligently controlled environmental system. Mol. Vis..

[20] Fariselli C., Giannaccare G., Fresina M., Versura P. (2018). Trehalose/hyaluronate eyedrop effects on ocular surface inflammatory markers and mucin expression in dry eye patients. Clin. Ophthalmol..

[21] Barabino S., Shen L., Chen L., Rashid S., Rolando M., Dana M.R. (2005). The Controlled-Environment Chamber: A New Mouse Model of Dry Eye. Investig. Opthalmology Vis. Sci..

[22] Hyun S.-W., Kim J., Park B., Jo K., Lee T.G., Kim J.S., Kim C.-S. (2019). Apricot Kernel Extract and Amygdalin Inhibit Urban Particulate Matter-Induced Keratoconjunctivitis Sicca. Molecules.

[23] Moon I., Kang H.G., Yeo A., Noh H., Kim H.C., Song J.S., Ji Y.W., Lee H.K. (2018). Comparison of Ocular Surface Mucin Expression After Topical Ophthalmic Drug Administration in Dry Eye-Induced Mouse Model. J. Ocul. Pharmacol. Ther..

[24] Lio C.T., Dhanda S., Bose T. (2020). Cluster Analysis of Dry Eye Disease Models Based on Immune Cell Parameters–New Insight Into Therapeutic Perspective. Front. Immunol..

[25] López-Miguel A., Tesón M., Martín-Montañez V., Enríquez-De-Salamanca A., Stern M.E., Calonge M., González-García M.J. (2014). Dry Eye Exacerbation in Patients Exposed to Desiccating Stress under Controlled Environmental Conditions. Am. J. Ophthalmol..

[26] Rahman M., Kim D.H., Park C.-K., Kim Y.H. (2021). Experimental Models, Induction Protocols, and Measured Parameters in Dry Eye Disease: Focusing on Practical Implications for Experimental Research. Int. J. Mol. Sci..

[27] Barabino S., Vilella E., Loreggian L., Marini S., Loretelli C., Spedicato G.A., Fiorina P., Rolando M. (2021). Efficacy of a New Tear Substitute Containing Hyaluronic Acid and a Low Dose of Hydrocortisone in Dry Eye Disease. Investig. Ophthalmol. Vis. Sci..

[28] Bucolo C., Lazzara F., Fidilio A., Platania C.B.M., Blanco A.R., Drago F. (2018). Effect of a New Ophthalmic Hydrocortisone and Sodium Hyaluronate Formulation on Two Experimental Dry Eye Models. Proceedings of the Acta Ophthalmologica.

[29] Cagini C., Muzi A., Castellucci G., Ragna G., Lupidi M., Alabed H.B.R., Pellegrino R.M. (2021). Kinetics of hydrocortisone sodium phosphate penetration into the human aqueous humor after topical application. Int. J. Clin. Pract..

[30] Chiambaretta F., Doan S., Labetoulle M., Rocher N., El Fekih L., Messaoud R., Khairallah M., Baudouin C., HA-trehalose Study Group (2017). A Randomized, Controlled Study of the Efficacy and Safety of a New Eyedrop Formulation for Moderate to Severe Dry Eye Syndrome. Eur. J. Ophthalmol..

[31] Laihia J., Kaarniranta K. (2020). Trehalose for Ocular Surface Health. Biomolecules.

[32] Mencucci R., Favuzza E., Decandia G., Cennamo M., Giansanti F. (2021). Hyaluronic Acid/Trehalose Ophthalmic Solution in Reducing Post-Cataract Surgery Dry Eye Signs and Symptoms: A Prospective, Interventional, Randomized, Open-Label Study. J. Clin. Med..

[33] Gipson I.K. (2016). Goblet cells of the conjunctiva: A review of recent findings. Prog. Retin. Eye Res..

[34] Murube J., Rivas L. (2003). Impression Cytology on Conjunctiva and Cornea in Dry Eye Patients Establishes a Correlation between Squamous Metaplasia and Dry Eye Clinical Severity. Eur. J. Ophthalmol..

[35] Willcox M.D., Argüeso P., Georgiev G., Holopainen J.M., Laurie G., Millar T.J., Papas E.B., Rolland J.P., Schmidt T.A., Stahl U. (2017). TFOS DEWS II Tear Film Report. Ocul. Surf..

[36] Mantelli F., Argüeso P. (2008). Functions of ocular surface mucins in health and disease. Curr. Opin. Allergy Clin. Immunol..

[37] Baudouin C., Rolando M., Del Castillo J.M.B., Messmer E.M., Figueiredo F.C., Irkec M., Van Setten G., Labetoulle M. (2019). Reconsidering the central role of mucins in dry eye and ocular surface diseases. Prog. Retin. Eye Res..

[38] Henriksson J.T., de Paiva C., Farley W., Pflugfelder S.C., Burns A.R., Bergmanson J.P.G. (2013). Morphologic Alterations of the Palpebral Conjunctival Epithelium in a Dry Eye Model. Cornea.

[39] Corrales R.M., Narayanan S., Fernández I., Mayo A., Galarreta D.J., Fuentes-Páez G., Chaves F.J., Herreras J.M., Calonge M., Fernandez I. (2011). Ocular Mucin Gene Expression Levels as Biomarkers for the Diagnosis of Dry Eye Syndrome. Investig. Opthalmology Vis. Sci..

[40] Perez V.L., Stern M.E., Pflugfelder S.C. (2020). Inflammatory basis for dry eye disease flares. Exp. Eye Res..

[41] Kessal K., Liang H., Rabut G., Daull P., Garrigue J.-S., Docquier M., Parsadaniantz S.M., Baudouin C., Brignole-Baudouin F. (2018). Conjunctival Inflammatory Gene Expression Profiling in Dry Eye Disease: Correlations with HLA-DRA and HLA-DRB1. Front. Immunol..

[42] Versura P., Profazio V., Schiavi C. (2011). Campos E Hyperosmolar Stress Upregulates HLA-DR Expression in Human Conjunctival Epithelium in Dry Eye Patients and in Vitro Models. Investig. Ophthalmol. Vis. Sci..

[43] Brignole-Baudouin F., Riancho L., Ismail D., Deniaud M., Amrane M., Baudouin C. (2017). Correlation Between the Inflammatory Marker HLA-DR and Signs and Symptoms in Moderate to Severe Dry Eye Disease. Investig. Opthalmol. Vis. Sci..

[44] Li Z., Woo J.M., Chung S.W., Kwon M.-Y., Choi J.-S., Oh H.-J., Yoon K.-C. (2013). Therapeutic Effect of Topical Adiponectin in a Mouse Model of Desiccating Stress–Induced Dry Eye. Investig. Opthalmol. Vis. Sci..

[45] Contreras-Ruiz L., Ghosh-Mitra A., Shatos M.A., Dartt D.A., Masli S. (2013). Modulation of Conjunctival Goblet Cell Function by Inflammatory Cytokines. Mediat. Inflamm..

